# Supplementary material for: Individual differences show that only some bats can cope with noise-induced masking and distraction
Source: PeerJ. 2020 Dec 18;8:e10551. doi: 10.7717/peerj.10551 (PMC7751433; doi:10.7717/peerj.10551)
Supplement: Supplemental Information 1 — Model output show the estimated differences in trial duration (relative to silence control trials) for the three noise treatments, ripple height, the number of days the bat was in the experiment, and the interaction between each noise treatment and ripple height, separately for each bat. Data were analyzed with inverse-Gaussian distribution and identity link function. Noise treatments have been abbreviated here (as compared to the text) to reduce visual clutter (smooth non-overlapping noise = “Non-overlap”; smooth-overlapping noise = “Smooth-overlap”; sparse-overlapping noise = “Sparse-overlap”). [file peerj-08-10551-s001.docx]

| **Bat** | **Variable** | **Estimate** | **SE** | **Z value** | **p value** |
| --- | --- | --- | --- | --- | --- |
| A | (Intercept) | 2.973 | 0.073 | 40.629 | <0.001 |
| A | Non-overlap | -0.241 | 0.116 | -2.085 | 0.14 |
| A | Smooth-overlap | -0.388 | 0.114 | -3.418 | 0.004 |
| A | Sparse-overlap | -0.135 | 0.125 | -1.080 | 0.731 |
| A | Ripple height | 0.010 | 0.005 | 1.953 | 0.189 |
| A | Day of experiment | 0.096 | 0.031 | 3.041 | 0.008 |
| A | Non-overlap:Ripple height | 0.006 | 0.008 | 0.700 | 0.929 |
| A | Smooth-overlap:Ripple height | 0.023 | 0.009 | 2.594 | 0.039 |
| A | Sparse-overlap:Ripple height | 0.009 | 0.009 | 0.976 | 0.797 |
| B | (Intercept) | 2.512 | 0.061 | 40.855 | <0.001 |
| B | Non-overlap | -0.046 | 0.101 | -0.454 | 0.985 |
| B | Smooth-overlap | 0.074 | 0.109 | 0.676 | 0.937 |
| B | Sparse-overlap | 0.086 | 0.103 | 0.840 | 0.871 |
| B | Ripple height | -0.015 | 0.003 | -4.382 | <0.001 |
| B | Day of experiment | -0.057 | 0.026 | -2.188 | 0.111 |
| B | Non-overlap:Ripple height | 0.006 | 0.006 | 0.924 | 0.828 |
| B | Smooth-overlap:Ripple height | 0.001 | 0.006 | 0.226 | 0.999 |
| B | Sparse-overlap:Ripple height | 0.000 | 0.006 | 0.012 | 1 |
| C | (Intercept) | 2.520 | 0.064 | 39.477 | <0.001 |
| C | Non-overlap | -0.130 | 0.109 | -1.193 | 0.654 |
| C | Smooth-overlap | -0.190 | 0.116 | -1.644 | 0.347 |
| C | Sparse-overlap | 0.644 | 0.133 | 4.844 | <0.001 |
| C | Ripple height | 0.009 | 0.004 | 1.944 | 0.192 |
| C | Day of experiment | 0.035 | 0.029 | 1.199 | 0.65 |
| C | Non-overlap:Ripple height | 0.020 | 0.008 | 2.394 | 0.066 |
| C | Smooth-overlap:Ripple height | 0.040 | 0.010 | 4.119 | <0.001 |
| C | Sparse-overlap:Ripple height | -0.004 | 0.009 | -0.485 | 0.981 |
| D | (Intercept) | 2.414 | 0.079 | 30.400 | <0.001 |
| D | Non-overlap | -0.222 | 0.114 | -1.941 | 0.196 |
| D | Smooth-overlap | -0.439 | 0.110 | -3.996 | <0.001 |
| D | Sparse-overlap | -0.173 | 0.133 | -1.299 | 0.578 |
| D | Ripple height | -0.010 | 0.005 | -2.154 | 0.122 |
| D | Day of experiment | 0.051 | 0.032 | 1.578 | 0.387 |
| D | Non-overlap:Ripple height | -0.014 | 0.006 | -2.309 | 0.081 |
| D | Smooth-overlap:Ripple height | -0.002 | 0.006 | -0.234 | 0.999 |
| D | Sparse-overlap:Ripple height | 0.016 | 0.009 | 1.800 | 0.258 |
