# Supplementary material for: Individual differences show that only some bats can cope with noise-induced masking and distraction
Source: PeerJ. 2020 Dec 18;8:e10551. doi: 10.7717/peerj.10551 (PMC7751433; doi:10.7717/peerj.10551)
Supplement: Supplemental Information 2 — Model output shows differences in aborted trials (relative to silence control trials) for the three noise treatments, ripple height, and the day of the experiment. Data were analyzed with a binomial distribution and logit link function. Noise treatments have been abbreviated here (as compared to the text) to reduce visual clutter (smooth non-overlapping noise = “Non-overlap”; smooth-overlapping noise = “Smooth-overlap”; sparse-overlapping noise = “Sparse-overlap”). [file peerj-08-10551-s002.docx]

| **Bat** | **Variable** | **Estimate** | **SE** | **Z value** | **p value** |
| --- | --- | --- | --- | --- | --- |
| A | (Intercept) | -3.594 | 0.326 | -11.013 | <0.001 |
| A | Non-overlap | -0.261 | 0.466 | -0.560 | 0.968 |
| A | Smooth-overlap | 0.487 | 0.381 | 1.279 | 0.592 |
| A | Sparse-overlap | 0.669 | 0.393 | 1.700 | 0.311 |
| A | Ripple height | 0.038 | 0.013 | 3.000 | 0.012 |
| A | Day of experiment | -0.086 | 0.169 | -0.507 | 0.977 |
| B | (Intercept) | -4.527 | 0.413 | -10.970 | <0.001 |
| B | Non-overlap | 1.790 | 0.449 | 3.987 | <0.001 |
| B | Smooth-overlap | 1.599 | 0.410 | 3.898 | <0.001 |
| B | Sparse-overlap | 2.467 | 0.476 | 5.180 | <0.001 |
| B | Ripple height | 0.051 | 0.010 | 4.977 | <0.001 |
| B | Day of experiment | -0.217 | 0.194 | -1.119 | 0.705 |
| C | (Intercept) | -3.143 | 0.313 | -10.041 | <0.001 |
| C | Non-overlap | 0.878 | 0.298 | 2.950 | 0.012 |
| C | Smooth-overlap | 1.776 | 0.424 | 4.188 | <0.001 |
| C | Sparse-overlap | 0.439 | 0.402 | 1.093 | 0.724 |
| C | Ripple height | 0.009 | 0.011 | 0.806 | 0.887 |
| C | Day of experiment | -0.600 | 0.168 | -3.570 | <0.001 |
| D | (Intercept) | -2.895 | 0.316 | -9.172 | <0.001 |
| D | Non-overlap | -0.633 | 0.528 | -1.198 | 0.65 |
| D | Smooth-overlap | -2.205 | 1.037 | -2.126 | 0.129 |
| D | Sparse-overlap | 1.364 | 0.356 | 3.829 | <0.001 |
| D | Ripple height | -0.023 | 0.017 | -1.341 | 0.548 |
| D | Day of experiment | -0.096 | 0.169 | -0.567 | 0.966 |
