# Supplementary material for: Individual differences show that only some bats can cope with noise-induced masking and distraction
Source: PeerJ. 2020 Dec 18;8:e10551. doi: 10.7717/peerj.10551 (PMC7751433; doi:10.7717/peerj.10551)
Supplement: Supplemental Information 6 — Model output shows differences in call frequency centroid (relative to silence control trials) for the three noise treatments, ripple height, the day of the experiment, and the interaction between each noise treatment and ripple height. Data were analyzed with log-normal (log-transformed Gaussian) distribution and identity link function. The mean frequency centroid was 69.7 kHz (bats A: 72.1 kHz; B: 69.3 kHz; C: 69.2 kHz; D: 67.8 kHz). Bats A, B, and C all decreased the frequency centroid in overlapping noise by 1.0, 0.8, and 0.6 kHz respectively (A: t = − 6.9, p < 0.001; B: t = − 3.4, p = 0.004; C: t = -3.4, p = 0.004), while only bat A decreased the centroid in sparse noise (by 0.9 kHz; t = − 5.8, p < 0.001). Bat A also increased the frequency centroid non-overlapping noise by 0.6 kHz, while non-coping bats decreased the frequency centroid in this treatment by 0.9 kHz each (A: t = 4.0, p < 0.001; C: t = − 5.0, p < 0.001; D: t = − 2.5, p = 0.051). Noise treatments have been abbreviated here (as compared to the text) to reduce visual clutter (smooth non-overlapping noise = “Non-overlap”; smooth-overlapping noise = “Smooth-overlap”; sparse-overlapping noise = “Sparse-overlap”). [file peerj-08-10551-s006.docx]

| **Bat** | **Variable** | **Estimate** | **SE** | **Z value** | **p value** |
| --- | --- | --- | --- | --- | --- |
| A | (Intercept) | 4.282 | 0.001 | 3422.992 | <0.001 |
| A | Non-overlap | 0.008 | 0.002 | 3.973 | <0.001 |
| A | Smooth-overlap | -0.014 | 0.002 | -6.865 | <0.001 |
| A | Sparse-overlap | -0.013 | 0.002 | -5.772 | <0.001 |
| A | Ripple height | 0.000 | 0.000 | -0.635 | 0.95 |
| A | Day of experiment | -0.001 | 0.001 | -1.264 | 0.605 |
| A | Non-overlap:Ripple height | 0.000 | 0.000 | -0.277 | 0.998 |
| A | Smooth-overlap:Ripple height | 0.000 | 0.000 | -0.359 | 0.994 |
| A | Sparse-overlap:Ripple height | 0.000 | 0.000 | 1.053 | 0.75 |
| B | (Intercept) | 4.242 | 0.002 | 2076.127 | <0.001 |
| B | Non-overlap | 0.001 | 0.003 | 0.389 | 0.992 |
| B | Smooth-overlap | -0.012 | 0.004 | -3.404 | 0.004 |
| B | Sparse-overlap | -0.005 | 0.003 | -1.607 | 0.367 |
| B | Ripple height | 0.000 | 0.000 | -0.450 | 0.986 |
| B | Day of experiment | 0.001 | 0.001 | 0.920 | 0.83 |
| B | Non-overlap:Ripple height | 0.000 | 0.000 | -0.262 | 0.998 |
| B | Smooth-overlap:Ripple height | 0.000 | 0.000 | 0.850 | 0.866 |
| B | Sparse-overlap:Ripple height | 0.000 | 0.000 | -0.320 | 0.996 |
| C | (Intercept) | 4.241 | 0.002 | 2585.737 | <0.001 |
| C | Non-overlap | -0.013 | 0.003 | -4.981 | <0.001 |
| C | Smooth-overlap | -0.009 | 0.003 | -3.350 | 0.004 |
| C | Sparse-overlap | 0.005 | 0.003 | 1.895 | 0.213 |
| C | Ripple height | 0.000 | 0.000 | -1.620 | 0.358 |
| C | Day of experiment | -0.001 | 0.001 | -1.238 | 0.622 |
| C | Non-overlap:Ripple height | 0.000 | 0.000 | 1.422 | 0.49 |
| C | Smooth-overlap:Ripple height | 0.000 | 0.000 | 1.839 | 0.239 |
| C | Sparse-overlap:Ripple height | 0.000 | 0.000 | 0.935 | 0.821 |
| D | (Intercept) | 4.223 | 0.003 | 1219.687 | <0.001 |
| D | Non-overlap | -0.014 | 0.006 | -2.501 | 0.051 |
| D | Smooth-overlap | -0.007 | 0.007 | -1.019 | 0.771 |
| D | Sparse-overlap | -0.003 | 0.009 | -0.364 | 0.993 |
| D | Ripple height | 0.000 | 0.000 | -0.629 | 0.951 |
| D | Day of experiment | -0.006 | 0.002 | -4.006 | <0.001 |
| D | Non-overlap:Ripple height | 0.000 | 0.000 | 0.105 | 1 |
| D | Smooth-overlap:Ripple height | 0.000 | 0.000 | -0.112 | 1 |
| D | Sparse-overlap:Ripple height | 0.000 | 0.001 | -0.563 | 0.967 |
